# Probabilistic projections of increased heat stress driven by climate change

**DOI:** 10.1038/s43247-022-00524-4

**Published:** 2022-08-25

**Authors:** Lucas R. Vargas Zeppetello, Adrian E. Raftery, David S. Battisti

**Affiliations:** 1 Department of Earth and Planetary Sciences, Harvard University, Cambridge, MA, USA.; 2 Department of Atmospheric Sciences, University of Washington, Seattle, WA, USA.; 3 Department of Statistics, University of Washington, Seattle, WA, USA.

## Abstract

The Heat Index is a metric that quantifies heat exposure in human beings. Here, using probabilistic emission projections, we show that changes in the Heat Index driven by anthropogenic CO_2_ emissions will increase global exposure to dangerous environments in the coming decades. Even if the Paris Agreement goal of limiting global warming to 2 °C is met, the exposure to dangerous Heat Index levels will likely increase by 50–100% across much of the tropics and increase by a factor of 3–10 in many regions throughout the midlatitudes. Without emissions reductions more aggressive than those considered possible by our statistical projection, it is likely that by 2100, many people living in tropical regions will be exposed to dangerously high Heat Index values during most days of each typical year, and that the kinds of deadly heat waves that have been rarities in the midlatitudes will become annual occurrences.

The deadly heat wave that struck Cascadia in the summer of 2021 was just the latest in a series of similar events that have impacted major cities in the past decade. The impacts of very high temperatures on public health and agricultural systems are highly consequential; the impacts of climate change on heat waves stand to present even more daunting challenges. Extreme heat contributes to chronic illnesses and is associated with regular losses of outdoor labor time^1^, and an “adaptability limit” to extremely high temperatures has the potential to threaten the habitability of large swaths of Earth’s land surface if greenhouse gas emissions are not curtailed^2^.

How will global warming impact people’s exposure to these very high temperatures? While several studies have estimated the projected increase in heat exposure due to climate change^3-5^, probabilistic projections have not been published, partly because estimates of climate change impacts usually depend on deterministic climate models run with only a handful of greenhouse gas emissions scenarios. In this paper, we use probabilistic projections of anthropogenic CO_2_ concentrations and a novel scaling approach that connects these global projections to local changes in temperature and relative humidity to better quantify the change in exposure to dangerously high temperatures due to climate change.

Heat Index is a metric for quantifying heat stress on human beings that takes into account the effects of temperature and relative humidity on heat stress and condenses the information into a single number, expressed as a temperature. A Heat Index above 103°F is classified by the United States’ National Weather Service as “dangerous” because of the likely onset of heat cramps and heat exhaustion. A Heat Index above 124°F is classified as “extremely dangerous” and can lead to heat stroke, a condition with a high mortality-case ratio that can lead to death within a matter of hours^6^. Given the interest in temperature extremes driven by climate change^7-9^ and new research showing that warming has already caused some populations to experience heat stress that approaches the limit of survivability^10^, this paper will quantify the extent to which different regions across Earth’s land surface will experience “dangerous” and “extremely dangerous” environments as defined by Heat Index thresholds.

## Results

### Projections of heat stress.

**Fig. 1:**
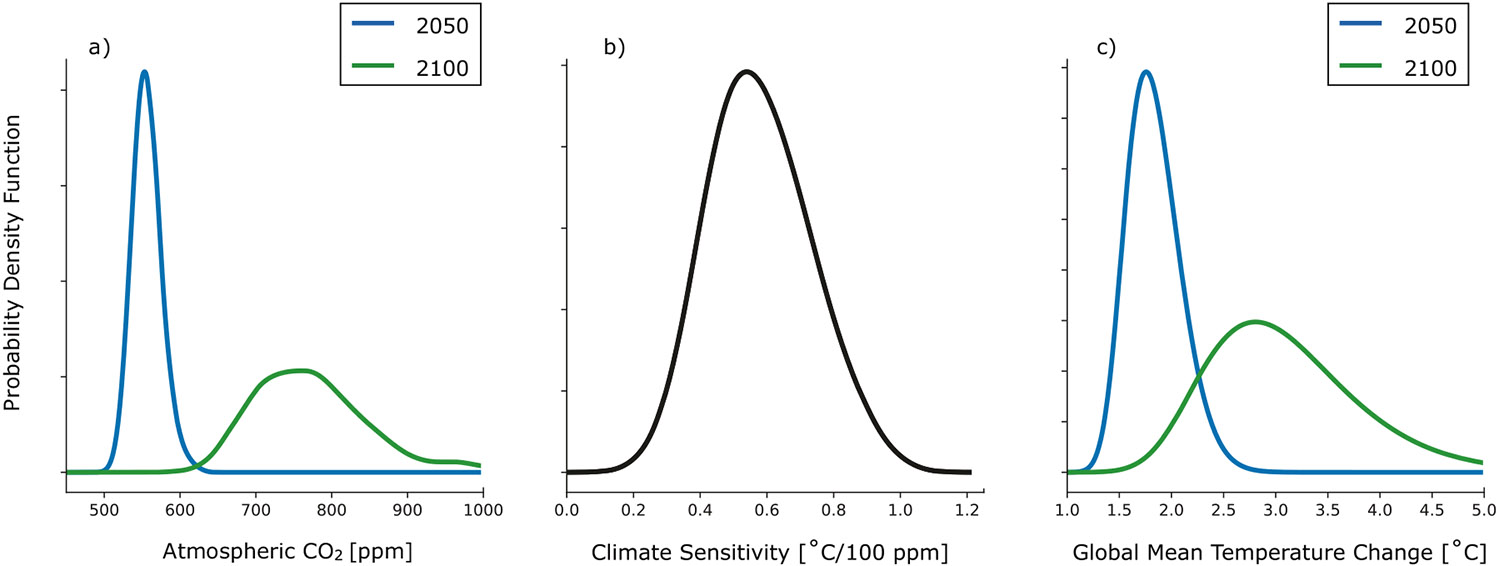
Projections of CO_2_ emissions and global mean temperature change through 2100. **a** shows probabilistic projections of atmospheric CO_2_ concentrations in 2050 and 2100. **b** shows a probability distribution of transient global climate sensitivity written in terms of °C warming per 100 ppm of atmospheric CO_2_ change. **c** shows the convolutions of the probability distributions in **a**, **b**, which yields a probability distribution of global mean temperature change (relative to the 1850–1900 baseline) in 2050 and 2100.

To quantify the degree to which climate change will increase human heat stress, we first need probabilistic projections of global mean temperature change driven by anthropogenic CO_2_ emissions. Figure 1a shows the probability density functions of atmospheric CO_2_ concentrations in the years 2050 and 2100. These were produced using a joint Bayesian model of change in population, Gross Domestic Product, and carbon intensity by country^11,12^.

While other greenhouse gases contribute to climate change on decadal to centennial timescales, atmospheric CO_2_ concentrations are highly correlated with global mean temperature change across a variety of climate change scenarios (see Table S1). We use linear best-fit regression to calculate the relationship between global mean temperature change and atmospheric CO_2_ concentrations in each of the 23 climate models that participated in the Coupled Model Intercomparison Project Phase 6 (CMIP6). The probability distribution of this linear relationship, which we refer to as the transient global climate sensitivity, is shown in Fig. 1b. The uncertainty in transient climate sensitivity is similar to that reported in other studies^13^ and reflects the different model physics and parameterizations that lead to various amounts of global warming across climate models forced by the same amount of anthropogenic emissions.

Anthropogenic emissions have already warmed the planet by roughly 1.0 °C as of 2000–2020 relative to 1850–1900 baseline^14,15^. Using the probability density functions in Fig. 1a, b along with the 1 °C warming already observed, we generate the probability density functions of global mean temperature change in 2050 and 2100, all relative to the 1850–1900 baseline used by the IPCC^16^, shown in Fig. 1c. For 2050, the [5, 50, 95] percentile changes in global mean temperature are [1.5, 1.8, 2.3] °C while for 2100 these percentiles are [2.1, 3.0, 4.3] °C. These projections indicate that there is only a 0.1% chance of limiting global average temperature change to the Paris Climate Agreement aspirational goal of 1.5 °C by 2100. Note that the full statistical model does not explicitly take into account the possibility of more aggressive policy actions such as negative emissions technologies and also does not consider overshoot strategies to achieve particular global warming targets.

**Fig. 2:**
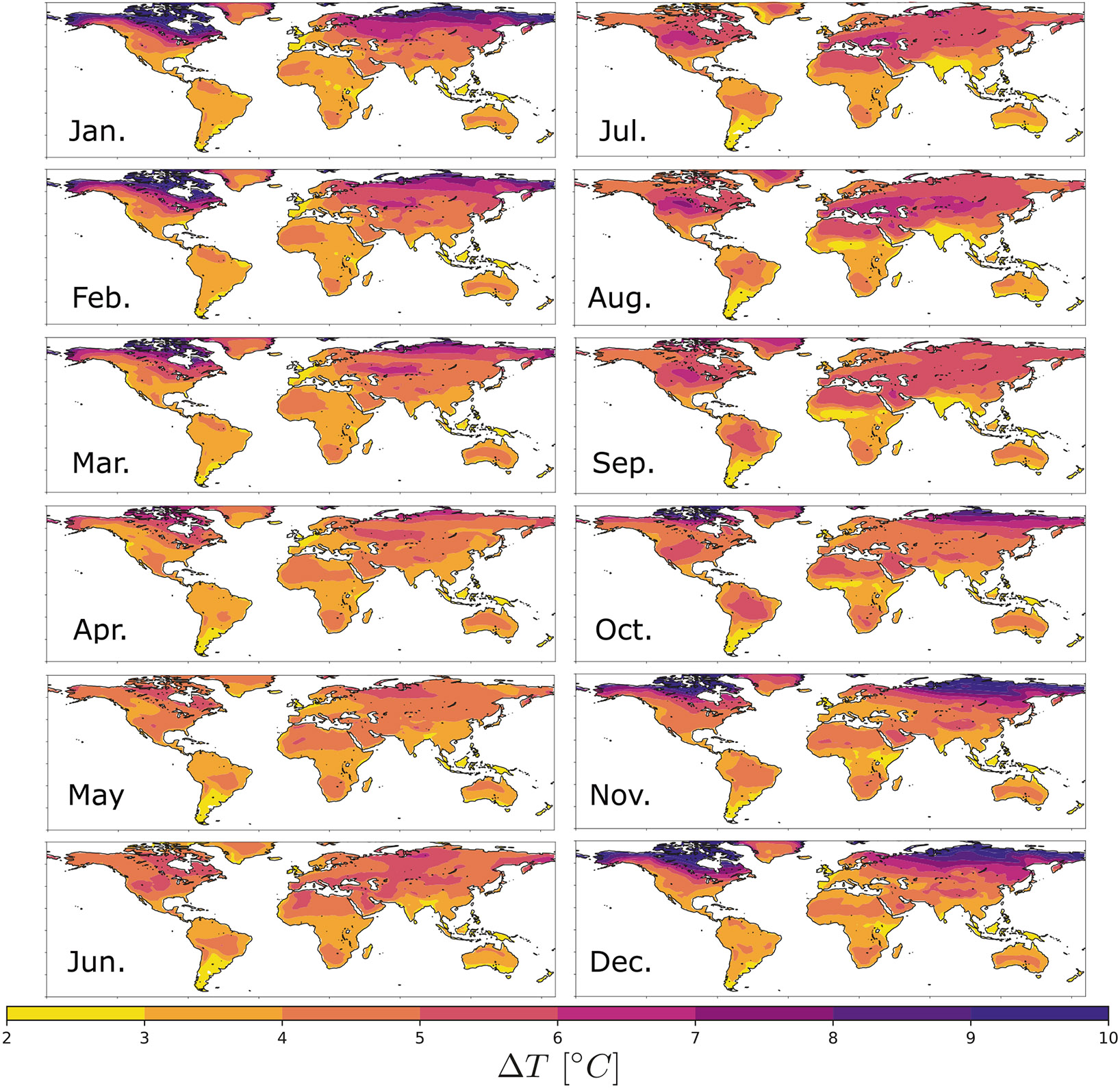
Local temperature change across months. Mean temperature change in each calendar month from the 50th percentile 2100 warming scenario (global mean temperature change of 3.0 °C).

To connect these probabilistic projections for the global mean temperature change to changes in local Heat Index, we calculate the ratio of local changes in temperature to global mean temperature change for each calendar month for each of the climate models and then averaged the results across the 23 climate models that we analyzed. This pattern scaling approach allows our probabilistic projections of global mean temperature change shown in Fig. 1c to be applied anywhere in space. The local mean temperature change for each month at each place in space is the product of the global mean temperature change in the scaling ratio $S_{T}$ shown in Fig. S1. The same procedure is applied to render the local change in relative humidity in each calendar month (see the scaling patterns $S_{\text{RH}}$ in Fig. S2). In Fig. 2, we show the regional changes in local temperature associated with the median global average temperature change (3.0 °C) projected for the end of this century. Changes over land regions are typically 5 °C, with greater increases in the Arctic.

To understand how the Heat Index changes with global warming, we first calculate the daily maximum Heat Index from 1979 to 1999 using daily observations of maximum temperature and monthly average observations of specific humidity (see Supplementary Methods). We then use six different scenarios of global mean temperature change that correspond to the [5,50,95] percentiles in 2050 and 2100 calculated from the PDF shown in Fig. 1c and the scaling patterns for temperature and relative humidity (Figs. S1 and S2) to calculate the change in the climatological mean temperature and relative humidity at each place in space for each calendar month in each of the six scenarios. This method of relating global to local mean temperature change takes advantage of a well-known pattern of temperature change seen across several generations of climate model ensembles^17^ and the (negative) correlation between global temperature and terrestrial relative humidity change^18^.

**Fig. 3:**
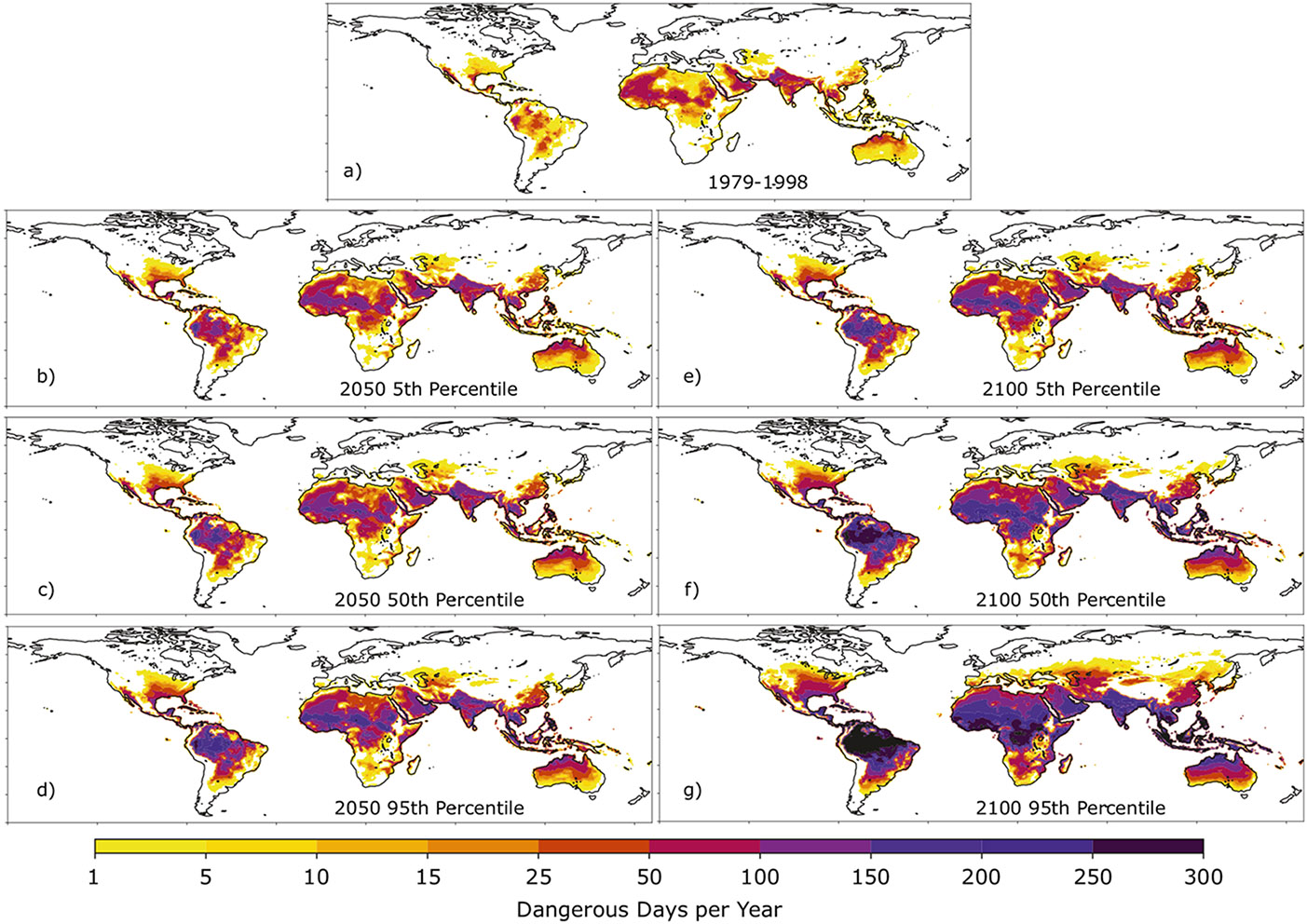
Projections of dangerous Heat Index values. **a** shows the average number of days per year when the dangerous Heat Index threshold was exceeded in the historical record (1979-1998). **b–g** show the same quantity under the various climate change scenarios noted in each panel.

**Fig. 4:**
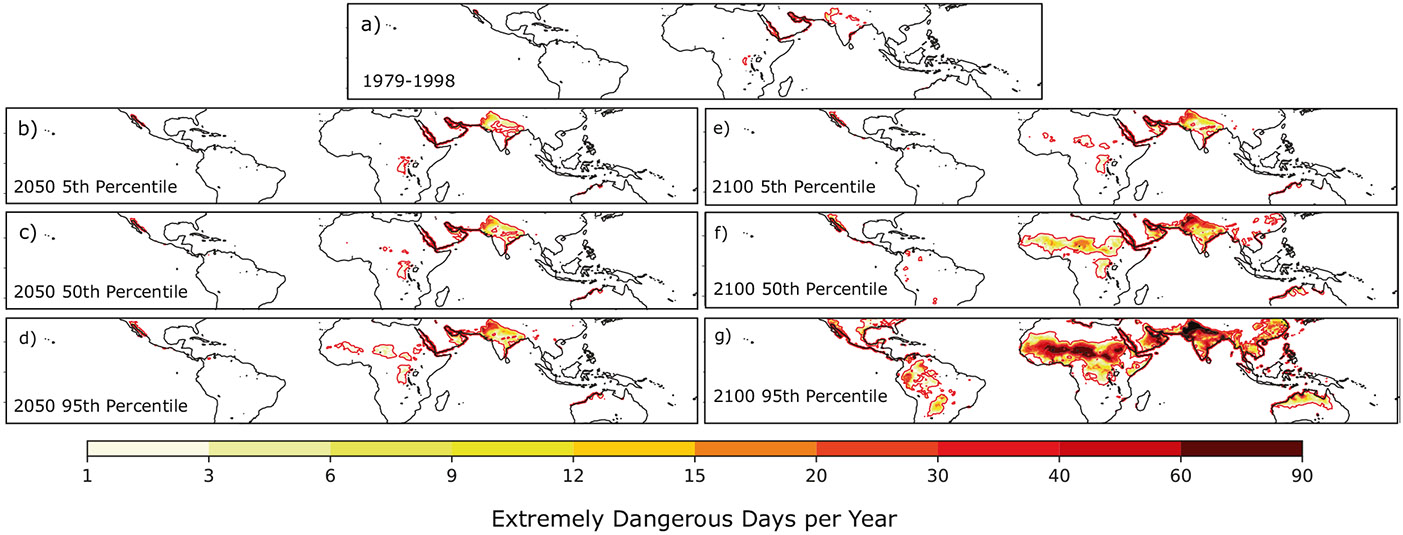
Projections of extremely dangerous Heat Index values. Same as Fig. 3a-g but for exceedances of the extremely dangerous Heat Index threshold. Red contours in all panels outline regions where the extremely dangerous Heat Index threshold is exceeded more than once per year on average.

We applied the relevant local changes in climatological temperature and relative humidity to the observed daily temperature and relative humidity (1979–1998) for each of the six climate change scenarios we considered, and then used these records as inputs to calculate the Heat Index according to the Rothfusz equation^19^. This procedure takes into account uncertainty in both projected CO_2_ emissions and climate sensitivity, but not the small uncertainty associated with regional uncertainty in global climate change projections (see “Methods” and Figs. S3-S5). Figures 3 and 4 show the average days per year where dangerous and extremely dangerous Heat Index thresholds are exceeded under six climate change scenarios, as well as in the observational record from 1979 to 1998 (see “Methods”).

Over the period 1979–1998, the dangerous Heat Index threshold was exceeded on roughly 5% of the days in each year in the tropics and subtropics (between 30°S and 30°N), and for 10–15% of the days in each year in subtropical Africa, the Indian subcontinent, and the Arabian peninsula (Fig. 3a). In the midlatitudes, the dangerous Heat Index threshold was exceeded less often; in many places, these exceedances represented extreme events that occurred less than once per year in the 20-year record we examined. Exceedances of the extremely dangerous Heat Index threshold were rare across the globe in the 1979–1998 record (see Fig. 4a). The most frequent exceedances of the extremely dangerous Heat Index threshold were concentrated in the coastal regions of the Arabian peninsula and Northern India and occurred between once and three times per year in the historical record.

The global warming scenarios present troubling projections of increasing heat stress driven by anthropogenic emissions. In the tropics and subtropics, where the dangerous Heat Index threshold was typically exceeded on less than 15% of the days in each year between 1979 and 1998, we project that, by 2050, many people living in these regions will likely experience dangerous Heat Index values on between one-quarter and one-half of all the days in each year (Fig. 3c). By 2100, the median projection is that most regions in the tropics and subtropics will exceed the dangerous Heat Index threshold on most of the days in each year (Fig. 3f). Many regions in the midlatitudes will experience dangerous Heat Index values on between 15 and 90 days each year—in some places, this represents an order of magnitude increase in the frequency of exposure to dangerous heat stress from the 1979–1998 period.

The Heat Index rarely exceeds the extremely dangerous threshold in the current climate (Fig. 4a). In the median projection for 2100 (Fig. 4f) extremely dangerous heat stress will be a regular feature of the climate in sub-Saharan Africa, parts of the Arabian peninsula, and much of the Indian subcontinent. The extremely dangerous Heat Index threshold is likely to be exceeded on more than 15 days in each year by the end of the century in these regions, this will likely require massive adaptation measures for a large number of people. In our 95th percentile projection, which corresponds to high emissions and high climate sensitivity (see Fig. 4g), the Heat Index will exceed extremely dangerous levels on between 15 and 25% of all days in each year in some tropical and subtropical regions.

### Chicago—a case study.

As an example from the midlatitudes, we turn to Chicago; a major urban center whose history illustrates the dangers of extremely high temperatures. An extreme drought swept the United States during the summer of 1988, causing billions of dollars in damages to the agriculture sector across the United States^20^. During the drought, Dr. James Hansen gave congressional testimony that human-induced increases in greenhouse gases could increase the probability of extreme events such as summer heat waves. These events marked a turning point in the public understanding of climate change.

During the 1988 heat wave, the Heat Index in Chicago was 5°F higher than average over the 1979–1989 period, but the 103°F “dangerous” threshold was never exceeded. Seven years later, in 1995, a heat wave devastated Chicago and caused nearly 800 excess deaths^21^. This event consisted of 4 consecutive days (July 12–15) when the Heat Index exceeded 100°F. Such an event (4 consecutive days of maximum Heat Index >100°F) occurred only twice in the 1979–1998 record, both times in 1995, but the other 1995 event had a lower average intensity and occurred later in the summer.

By randomly sampling 1000 scenarios of global mean temperature changes from the distribution shown in Fig. 1c and using the local scaling patterns for Chicago’s place in space, we quantify the average change to Chicago’s daily Heat Index record by 2100. To do this, we augmented the 1979–1998 record of temperature and relative humidity in the same manner as was done in Figs. 3 and 4 (see “Methods”).

The 1979–1998 record shows that a daily Heat Index of 100°F was not exceeded in 11 out of 20 years. The same 20-year record modified by the median projections of temperature and relative humidity changes for the end of this century has at least one exceedance of this threshold each year. Further, heat waves like the kind that Chicago experienced in 1995 are projected to become a regular occurrence by the end of the century in our median projection: two 4-day periods with daily maximum Heat Index >100°F were found in the 20-year historical record (1979–1998); our median projection shows 32 such events in a 20-year period at the end of this century. This 16-fold increase in the number of potentially dangerous heat waves points to the kind of societal adaptation required to combat these phenomena in the midlatitudes. This order of magnitude increase in the number of heat waves in a 20-year record is reflected in our median projection of the number of days per year where the dangerous Heat Index threshold is exceeded. In the 1979–1998 record, the dangerous threshold (103°F) was exceeded four times (all in 1995), while an average of 11 exceedances of this threshold each year is likely by 2100.

## Conclusions

We have developed probabilistic projections of the average number of days per year that the Heat Index will exceed “dangerous” and “extremely dangerous” levels. The temperature projections on which they are based take into account probabilistic projections of country-scale global CO_2_ emissions and the probability density function of global transient climate sensitivity. They show that global mean temperature change will likely approach 2 °C by 2050, with a median projection 1.8 °C, assuming that recent system dynamics do not change dramatically.

It is likely that, without major emissions reductions, large portions of the global tropics and subtropics would experience Heat Index levels higher than considered “dangerous” for a majority of the year by the end of the century. Without adaptation measures, this would greatly increase the incidence of heat-related illnesses and reduce outdoor working capacity in many regions where subsistence farming is important^22^. Regions where the extremely dangerous Heat Index threshold is almost never exceeded today will experience between one and fifteen days when the extremely dangerous Heat Index threshold is exceeded each year. We project that, by the end of the twenty-first century, these regions will include large portions of India and sub-Saharan Africa. According to the UN demographic projections for 2100, these regions are projected to include about 5.3 billion people by 2100, or about half the world’s population at that time. Note that these results are conditional on historical trends in Kaya parameters and do not explicitly take into account possible carbon cycle feedbacks.

In the midlatitudes where “dangerous” Heat Index exceedances are far less frequent than in the tropics, our case study of the Chicago heat wave of 1995 indicates how heat waves of similar intensity to those that have killed hundreds in major cities will go from outliers to relatively common occurrences. The health consequences of regular very high temperatures, particularly for the elderly, poor, and outdoor workers, would be profound and require a basic reorientation to the risks of extreme heat even in the midlatitudes.

## Methods

### Probabilistic carbon dioxide emissions projections.

We produced probabilistic projections of CO_2_ emissions to the end of the century using a joint Bayesian hierarchical model (BHM) of population change, GDP, and carbon intensity^11,12^. This is based on a country-specific version of the Kaya identity, which expresses carbon emissions as a product of population, GDP, and carbon intensity for each country in each year. Carbon intensity is defined as carbon emissions per unit of GDP, and GDP is expressed in terms of Purchasing Power Parity (PPP). The BHM is estimated using annual data from 1960 to 2015 on population, GDP, and carbon emissions for each country. Because of data limitations, some countries had to be omitted. The 152 countries used account for over 98% of both the world’s population and GDP.

The Bayesian hierarchical model was simulated forward to 2100 many times to obtain a large number of conditionally independent trajectories of future carbon emissions to 2100 from all countries considered. For each trajectory, the emissions from all these countries were added together to get a trajectory of future global emissions in each year to 2100. This yields a probability distribution of future global emissions in each year. Validation experiments have indicated the resulting predictive distributions to be well calibrated^11^. Following the Global Carbon Budget analysis,^23^ we use 1 ppm = 7.8 Gt CO_2_ as a conversion factor between the emissions projections and the atmospheric concentration change which takes into account the airborne fraction of carbon dioxide emissions and assumes no change in this value as atmospheric CO_2_ increases. We also use 412 ppm as our baseline value for 2020^24^.

### Estimating transient climate sensitivity.

We calculated the global mean temperature for each year from 2015 to 2100 using output from 23 models that participated in the Coupled Model Intercomparison Project Phase 6 (CMIP6) and were forced by four different emissions scenarios (SSP1-2.6, SSP2-4.5, SSP3-7.0, and SSP5-8.5). Table S1 shows the variance of annual average global mean temperature explained by the atmospheric CO_2_ concentrations. On average across models, the atmospheric CO_2_ concentrations in each of the SSP scenarios explain 93% of the variance in global mean temperature change. The relative linearity found in the projections for the next century allows us to simplify climate sensitivity as the slope of the best-fit linear regression line between atmospheric CO_2_ concentration and global mean temperature change. We calculated this slope for each model in Table S1 and used a kernel density estimator to generate the probability density function shown in Fig. 1b.

### Global and local climate change.

To connect global mean temperature changes to local changes in mean temperature and relative humidity, we used output from the SSP5-8.5 scenario and calculated the ratio of local changes between 2081–2100 and the past 20 years of the historical period (1996–2015) in temperature and relative humidity to the global mean temperature change for each calendar month and for each of models and then averaged the results over the 23 climate models we analyzed. We refer to these ratios as “scaling patterns” for temperature $S_{T}$ and relative humidity $S_{\text{RH}}$, and they are shown for each month in Figs. S1 and S2. While the mean warming pattern is known to be relatively consistent across climate models^17^ and the relationship between relative humidity changes over land and global mean temperature change has been detailed from observations^18^, there is still uncertainty in these pattern effects that is not captured by our analysis. We discuss this source of uncertainty in our projections after describing how we use these scaling patterns to project the Heat Index in various climate scenarios.

### Historical and future Heat Index.

Figures 3a and 4a show the average number of days per year in which the local Heat Index exceeded “dangerous” and “extremely dangerous” thresholds for the period 1979-1998. Analysis of the HadISD record shows that the “dangerous” Heat Index threshold of 103°F corresponds to Wet Bulb temperatures between 27–29 °C and the “extremely dangerous” Heat Index threshold of 124°F corresponds to roughly 33–35 °C (though the extreme nature of these events makes a robust comparison difficult^10^). As inputs to the Heat Index equation developed by Rothfusz^19^, we used daily values of maximum temperature from the Climate Prediction Center and monthly averaged values of specific humidity from ERA5. We then calculated daily relative humidity (the second input into the Rothfusz equation for Heat Index) by using the daily maximum temperature and the monthly averaged specific humidity. As a check on whether the use of monthly averaged (rather than daily) specific humidity leads to meaningful errors in the Heat Index, for several major sites across climate zones we calculated the Heat Index using the method described above with that calculated using daily temperature and specific humidity. The variance values in the daily Heat Index using the daily values of specific humidity were within 10% of those found using monthly average values of specific humidity. This is not surprising because variations in saturation-specific humidity driven by near-surface air temperature have a much larger impact on daily relative humidity than do daily variations in specific humidity^25,26^. However, future work should consider specific humidity variations and an external forcing on extreme events given the importance of so-called “humid heat waves” in the global tropics.

To project future changes in the distribution of Heat Index due to the projected changes in temperature and relative humidity due to anthropogenic warming, we first calculate the projected global mean temperature change corresponding to the year we wish to examine (Fig. 1c) and then subtract 0.6 °C to account for the fact that this amount of warming had occurred prior to the 1979–1998 period. We then multiply the global mean temperature change by the corresponding scaling patterns $S_{T}$ and $S_{\text{RH}}$ in Figs. S1 and S2 to determine the monthly mean changes in temperature and relative humidity expected at each place in space across the land surface. We then take these monthly changes in temperature and relative humidity at each place and add them to the daily record of maximum temperature and average relative humidity for the period 1979–1998 to obtain a daily 20-year record of Heat Index that corresponds to each particular climate scenario.

In effect, we assume a future 20-year period experiences the same daily weather as for the period 1979–1998, acting on top of a warmer and (in most regions) drier mean climate. We therefore circumvent the large biases in the natural variability simulated by climate models. These new 20-year Heat Index records are used to calculate the average number of days for which the Heat Index is projected to exceed the dangerous and extremely dangerous thresholds, shown in Figs. 3 and 4. For the Chicago case study, we sampled 1000 values of global mean temperature change from Fig. 1c and produced 1000 corresponding 20-year records using the pattern scaling values for Chicago’s place in space. The results quoted in that section are averages across these 1000 20-year records. Note that we are assuming that the particular pattern of variability of 1979–1998 is representative of future variability under the warming scenario.

### Uncertainty in the scaling patterns.

To quantify the uncertainty in our Heat Index estimates due to differences in the scaling patterns across climate models, Fig. S3 shows the standard deviation of the local differences in the temperature scaling patterns $S_{T}$ across the 23 climate models. The pattern scaling values in Fig. S1 are fairly consistent over land across CMIP6 models: values shown in Fig. S3 are <25% of those in Fig. S1, indicating that the ensemble mean patterns in Fig. S1 account for about 90% of the variance in local mean temperature change across models. Uncertainty in $S_{T}$ (relative to the multi-model-mean) tends to be greatest in places where vegetation dynamics make up a large portion of inter-model spread^27^. Similarly, Fig. S4 shows the standard deviation in the relative humidity pattern scaling values $S_{\text{RH}}$ across the 23 climate models. The model uncertainties in relative humidity changes tend to be largest in regions with the greatest ensemble mean changes in relative humidity, particularly over the Amazon rain forest and in the Sahel (two regions where vegetation dynamics are likely crucial to determining changes to atmospheric humidity).

To quantify the extent to which these model differences in the pattern scaling values $S_{T}$ and $S_{\text{RH}}$ contribute to uncertainty in the average number of days per year where the dangerous Heat Index threshold is exceeded, we perform two experiments using the 2100 median projection for global mean temperature change. In the first (EXP1), we add $1\sigma (S_{T})$ to the temperature pattern scaling values $S_{T}$ for each calendar month and subtract $1\sigma (S_{\text{RH}})$ from the relative humidity scaling values $S_{\text{RH}}$ for each month: higher mean temperatures driving to lower values of relative humidity has support from observations^18^ and has been found to be true regionally across climate models^28^. In the second experiment (EXP2), we reverse the sign of both modifications and subtract $1\sigma (S_{T})$ from the temperature scaling patterns for each calendar month while adding $1\sigma (S_{\text{RH}})$ to the relative humidity scaling patterns. These two modifications have opposing effects on the Heat Index in both experiments; warmer temperatures act to increase the Heat Index while lower values of relative humidity tend to decrease it (and vice versa). Figure S5 shows the ratio of the number of days per year in a 20-year record that the dangerous Heat Index threshold is exceeded in our uncertainty experiments to the number of dangerous exceedances from Fig. 3f, which uses the multi-model-mean values of $S_{T}$ and $S_{\text{RH}}$.

Figure S5 shows that uncertainty in the pattern scaling effects has little bearing on the number of dangerous Heat Index threshold exceedances; most of the global land surface (particularly the tropics) experiences less than a 5% change in both experiments. Importantly, the results of these experiments are not homogenous across space; i.e., a warmer and drier world does not always imply more heat stress. In the Central U.S., for example, the summertime uncertainty in relative humidity change is the dominant factor in the uncertainty, and the potential drying effects on Heat Index outweigh the impacts of higher temperatures, while across southern Europe, the effect of higher temperatures is predominant. While the relative humidity projections are particularly uncertain, Fig. S5 shows that the differences between the two experiments are relatively small compared to the 50–100% increases in exposure to dangerous Heat Index levels projected for the tropics and subtropics at the end of the century, and the 3–10-fold increase projected in the midlatitudes.

## Supplementary Material

Supplementary Information

## Data Availability

Source information from the figures can be found at https://doi.org/10.5281/zenodo6857267. Climate model outputs were obtained from the ESGF node (https://esgf.llnl.gov). Daily temperatures were obtained from the CPC Global Unified Temperature Dataset (https://psl.noaa.gov/data/gridded/data.cpc.globaltemp.html). ERA5 data were obtained from the ECMWF portal (https://www.ecmwf.int/en/forecasts/datasets/reanalysis-datasets/era5).

## References

[1] Spector J & Sheffield P Re-evaluating occupational heat stress in a changing climate. Ann. Occup. Hyg 58, 936–942 (2014). 25261455 10.1093/annhyg/meu073 PMC4481564

[2] Sherwood SC & Huber M An adaptability limit to climate change due to heat stress. Proc. Natl Acad. Sci. USA 107, 9552–9555 (2010). 20439769 10.1073/pnas.0913352107 PMC2906879

[3] Willett KM & Sherwood SC Exceedance of heat index thresholds for 15 regions under a warming climate using the wet-bulb globe temperature. Int. J. Climatol 32, 161–177 (2012).

[4] Dunne J, Stouffer R & John J Reductions in labour capacity from heat stress under climate warming. Nat. Clim. Change 3, 563–566 (2013).

[5] Schwingshackl C, Sillmann J, Vicedo-Cabrera AM, Sandstad M & Aunan K Heat stress indicators in CMIP6: estimating future trends and exceedances of impact-relevant thresholds. Earths Future 9, e2020EF001885 (2021).

[6] Kovats R & Hajat S Heat stress and public health: a critical review. Annu. Rev. Public Health 29, 41–55 (2008). 18031221 10.1146/annurev.publhealth.29.020907.090843

[7] Easterling DR Climate extremes: observations, modeling, and impacts. Science 289, 2068–2074 (2000). 11000103 10.1126/science.289.5487.2068

[8] Huybers P, McKinnon KA, Rhines A & Tingley M U.S. daily temperatures: the meaning of extremes in the context of nonnormality. J. Clim 27, 73684–7384 (2014).

[9] Donat MG, Pitman AJ & Seneviratne SI Regional warming of hot extremes accelerated by surface energy fluxes. Geophys. Res. Lett 44, 7011–7019 (2017).

[10] Raymond C, Matthews T & Horton RM The emergence of heat and humidity too severe for human tolerance. Sci. Adv 6, eaaw1838 (2020). 32494693 10.1126/sciadv.aaw1838 PMC7209987

[11] Raftery A, Zimmer A & Frierson D Less than 2°C warming by 2100 unlikely. Nat. Clim. Change 7, 637–641 (2017). 10.1038/nclimate3352 PMC6070153 30079118

[12] Liu P & Raftery A Country-based rate of emissions reductions should increase by 80% beyond nationally determined contributions to meet the 2°C target. Commun. Earth Environ 2, 29 (2021). 33899003 10.1038/s43247-021-00097-8 PMC8064561

[13] Sherwood SC An assessment of earth’s climate sensitivity using multiple lines of evidence. Reviews of Geophysics 58, e2019RG000678 (2020). 10.1029/2019RG000678 PMC7524012 33015673

[14] NOAA. National Centers for Environmental Information. State of the Climate: Global Climate Report for Annual 2020. Technical Report. https://www.ncdc.noaa.gov/sotc/global/202013 (2021).

[15] IPCC. Climate Change 2021: The Physical Science Basis. Contribution of Working Group I to the Sixth Assessment Report of the Intergovernmental Panel on Climate Change (Cambridge University Press, 2021).

[16] IPCC. Global Warming of 1.5 °C. An IPCC Special Report on the Impacts of Global Warming of 1.5 °C Above Pre-industrial Levels and Related Global Greenhouse Gas Emissions Pathways, in the Context of Strengthening the Global Response to the Threat of Climate Change, Sustainable Development, and Efforts to Eradicate Poverty (World Meteorological Organization, 2018).

[17] Knutti R & Sedláček J Robustness and uncertainties in the new cmip5 climate model projections. Nat. Clim. Change 3, 369–373 (2013).

[18] Byrne MP & O’Gorman PA Trends in continental temperature and humidity directly linked to ocean warming. Proc. Natl Acad. Sci. USA 115, 4863–4868 (2018). 29686095 10.1073/pnas.1722312115 PMC5948989

[19] Rothfusz LP The Heat Index Equation (or, More Than You Ever Wanted to Know About Heat Index). Technical Report (National Weather Service, 1990).

[20] Trenberth KE, Branstator GW & Arkin PA Origins of the 1988 North American drought. Science 242, 1640–1645 (1988). 17730574 10.1126/science.242.4886.1640

[21] Changnon SA, Kunkel KE & Reinke BC Impacts and responses to the 1995 heat wave: a call to action. Bull. Am. Meteorol. Soc 77, 1497–1506 (1996).

[22] Rapsomanikis G. The Economic Lives of Smallholder Farmers: An Analysis Based on Household Data From Nine Countries. Technical Report (FAO, 2015).

[23] Friedlingstein P. Global carbon budget 2020. Earth Syst. Sci. Data 12, 3269–3340 (2020).

[24] Dlugokencky E & Tans P Globally averaged marine surface data. Technical Report, NOAA Global Marine Laboratory. https://gml.noaa.gov/ccgg/trends/gl_data.html (2021).

[25] van Heerwaarden CC, de Arellano JV-G, Gounou A, Guichard F & Couvreux F Understanding the daily cycle of evapotranspiration: a method to quantify the influence of forcings and feedbacks. J. Hydrometeorol 11, 1405–1422 (2010).

[26] Vargas Zeppetello L, Battisti D & Baker M The origin of soil moisture evaporation “regimes”. J. Clim 32, 6939–6960 (2019).

[27] Zarakas CM, Swann ALS, Laguë MM, Armour KC & Randerson JT Plant physiology increases the magnitude and spread of the transient climate response to CO_2_ in CMIP6 earth system models. J. Clim 33, 8561–8578 (2020).

[28] Vargas Zeppetello LR & Battisti DS Projected increases in monthly midlatitude summertime temperature variance over land are driven by local thermodynamics. Geophys. Res. Lett 47, e2020GL090197 (2020).

