# Supplementary material for: Probabilistic projections of increased heat stress driven by climate change
Source: Commun Earth Environ. Author manuscript; Available in PMC 2024 Oct 17. (PMC11485542; doi:10.1038/s43247-022-00524-4)
Supplement: Supplementary Information [file NIHMS2024676-supplement-Supplementary_Information.pdf]

Supplementary information for "Probabilistic Projections of Increased Heat Stress Driven By Climate Change"

Lucas R. Vargas Zeppetello,[1] Adrian E. Raftery,[2] David S. Battisti[3]

[1]Department of Earth and Planetary Sciences, Harvard University

[2]Department of Statistics, University of Washington

[3] Department of Atmospheric Sciences, University of Washington

*To whom correspondence should be addressed; E-mail: lzeppetello@fas.harvard.edu.

Supplementary Table and Figures

Model Name	r^2	Model Name	r^2	Model Name	r^2	Model Name	r^2
ACCESS-CM2	0.93	CESM2	0.96	GISS-E2-1-G	0.89	MIROC6	0.91
AWI-CM-1-1-MR	0.95	CMCC-CM2-SR5	0.87	IITM-ESM	0.94	MPI-ESM1-2-LR	0.95
BCC-CSM2-MR	0.93	CNRM-CM6-1	0.96	INM-CM4-8	0.96	MRI-ESM2-0	0.95
CAMS-CSM1-0	0.91	EC-Earth3	0.94	IPSL-CM6A	0.96	TaiESM1	0.88
CanESM5	0.96	FGOALS-f3-L	0.87	KACE-1-0-G	0.94	UKESM1-0-LL	0.95
CAS-ESM2-0	0.87	GRDL-ESM4	0.94	MCM-UA-1-0	0.94	Multi-Model-Mean	0.93

Table S1: The 23 general circulation models analyzed in this study and the proportion of the variance in annual mean global mean temperature explained by atmospheric CO_2 concentrations (r^2).

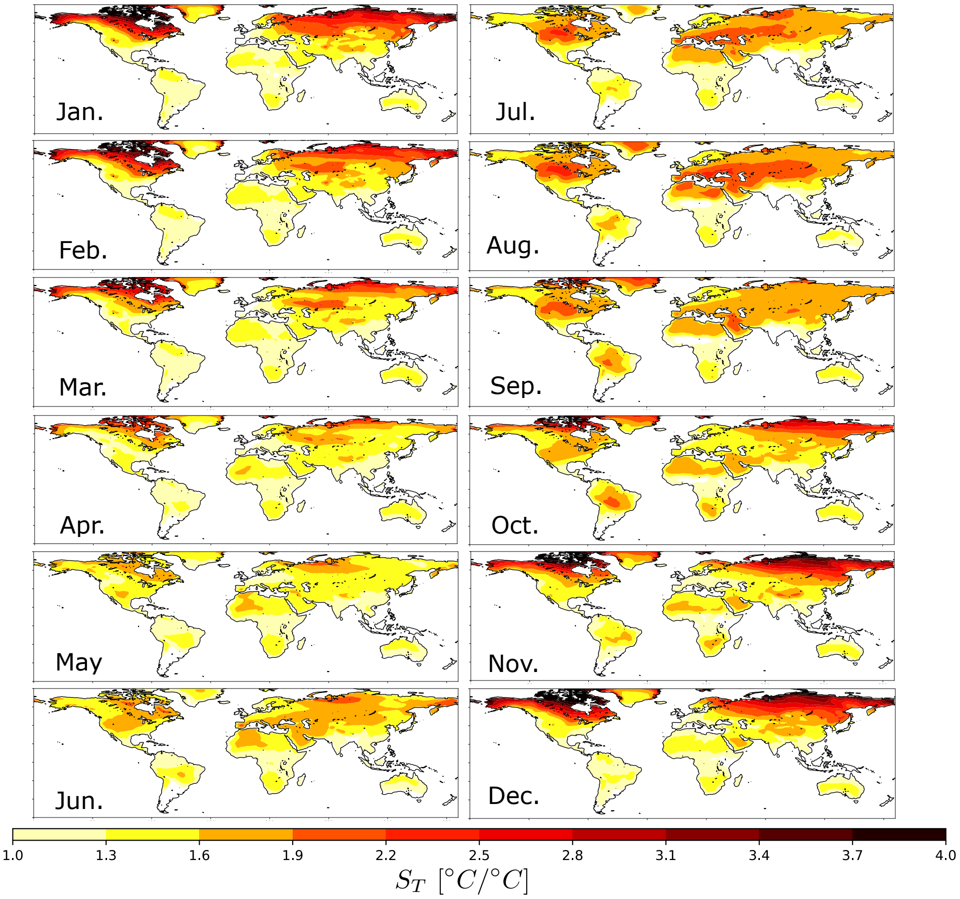

Figure S1: Scaling patterns of local mean temperature change to global mean temperature change S_T from 23 CMIP6 models calculated from the SSP5-8.5 scenario and the historical simulations. Results are shown for each month of the year.

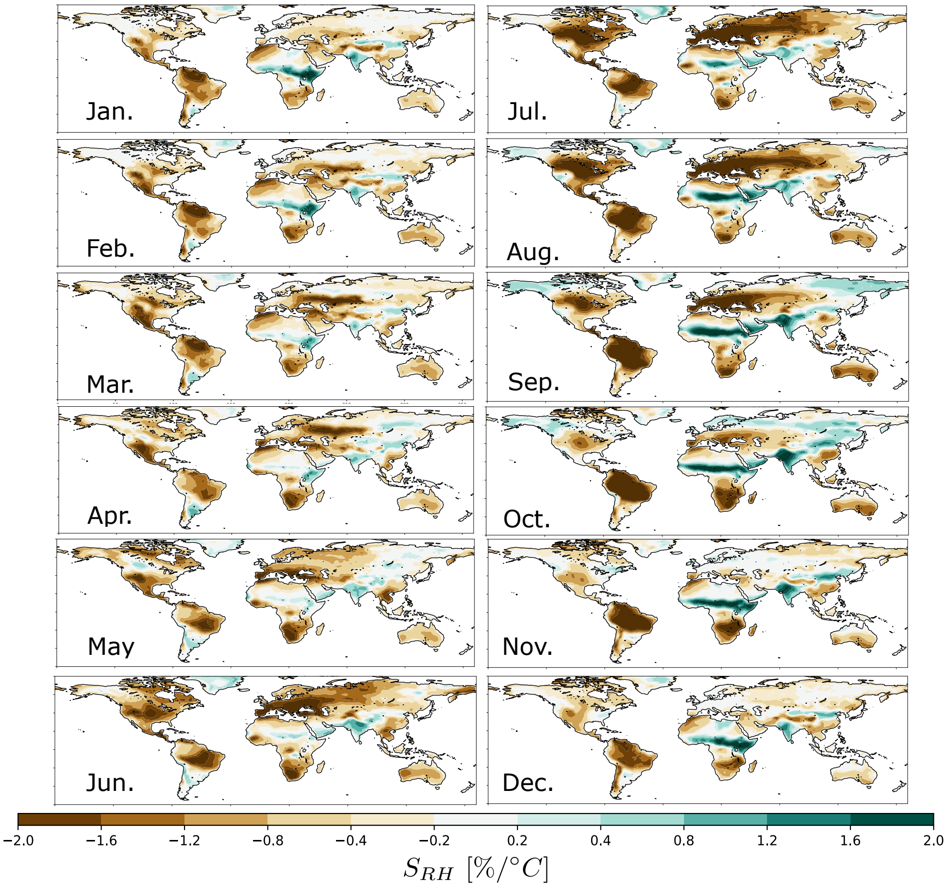

Figure S2: Same as Fig. S1 but for the scaling patterns of local mean relative humidity change to global mean temperature change S_{RH}.

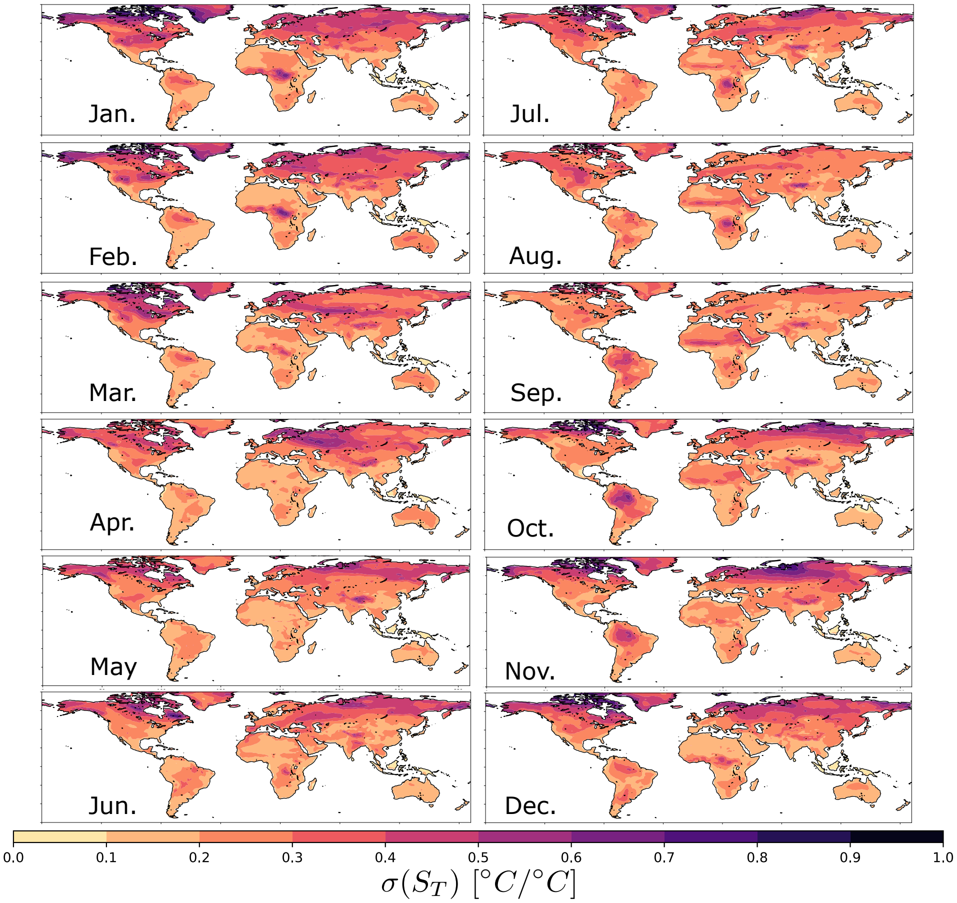

Figure S3: Standard deviation of the scaling patterns S_T across 23 climate models for each month in the calendar year.

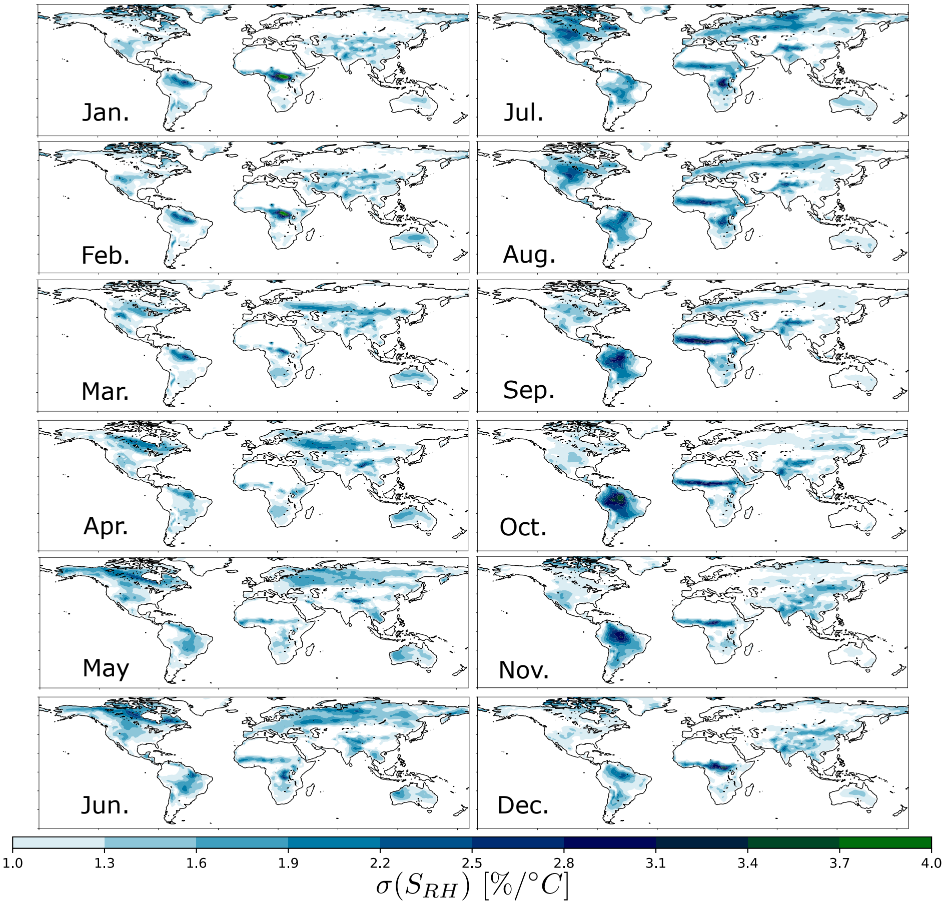

Figure S4: Standard deviation of the scaling patterns S_{RH} across 23 climate models for each month in the calendar year.

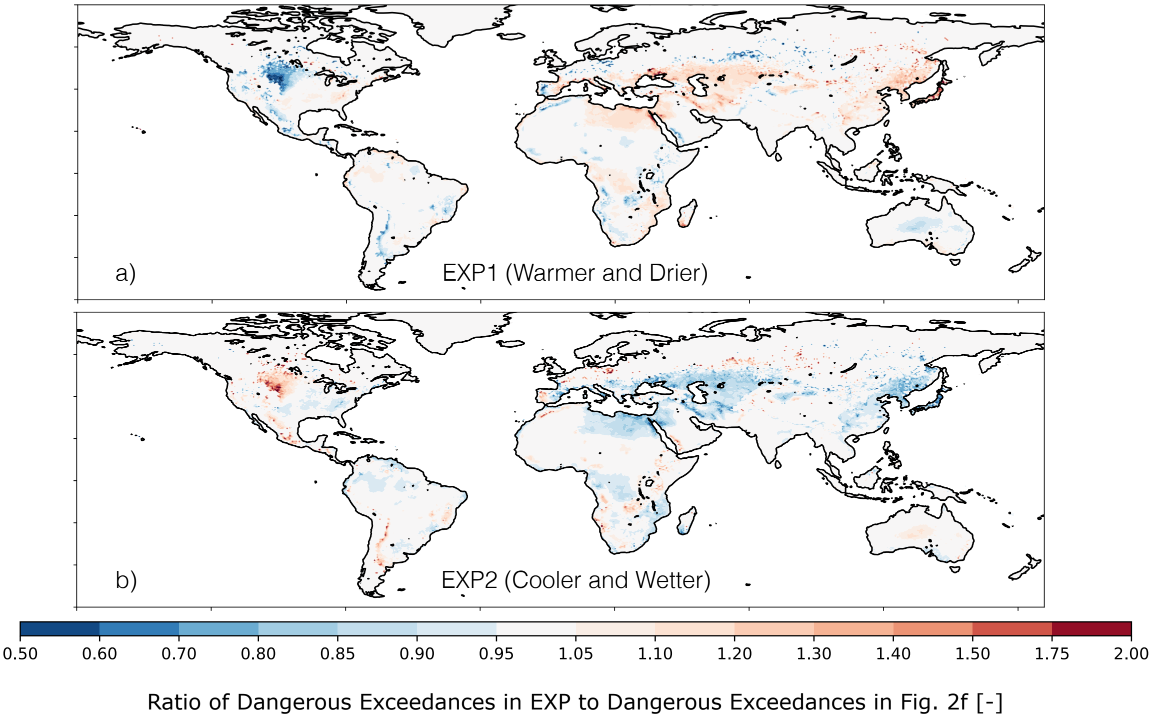

Figure S5: Ratio of dangerous exceedances per year in uncertainty experiments to the number of dangerous exceedances per year in Fig. 3f of the main paper, which uses the multi-model-mean scaling patterns Panel (a) shows the results from the warmer and drier experiment (EXP1) and panel (b) shows results from the cooler and wetter experiment (EXP2).
